# Overexpression of *DoUGP* Enhanced Biomass and Stress Tolerance by Promoting Polysaccharide Accumulation in *Dendrobium officinale*

**DOI:** 10.3389/fpls.2020.533767

**Published:** 2020-11-16

**Authors:** Ji Chen, Li Wang, Huan Liang, Xiaowan Jin, Jian Wan, Fan Liu, Ke Zhao, Jin Huang, Mengliang Tian

**Affiliations:** ^1^Agronomy College, Sichuan Agricultural University, Chengdu, China; ^2^College of Ecology and Environment, Chengdu University of Technology, Chengdu, China; ^3^Institute for New Rural Development, Sichuan Agricultural University, Yaan, China

**Keywords:** *Dendrobium officinale*, polysaccharide, *DoUGP*, biomass, stress resistance

## Abstract

Uridine diphosphate glucose pyrophosphorylase (UDP-glucose pyrophosphorylase, UGPase), as one of the key enzymes in polysaccharide synthesis, plays important roles in the growth and development of plants. In this study, the *DoUGP* gene of *Dendrobium officinale* was overexpressed. The expression of *DoUGP* and genes playing roles in the same and other saccharide synthesis pathways was determined, and the total soluble polysaccharide was also tested in wild-type and transgenic seedlings. We also performed freezing and osmotic stress treatments to determine whether overexpression of *DoUGP* could influence stress resistance in transgenic seedlings. Results showed that mRNA expression levels of *DoUGP* and its metabolic upstream and downstream genes in the transgenic seedlings were increased compared to the expression of these genes in wild-type seedlings. Additionally, most *CSLA* genes involved in the biosynthesis of mannan polysaccharides were significantly upregulated. The total polysaccharide and mannose content of transgenic seedlings were increased compared to the content of wild type, and enhanced stress tolerance was found in the overexpressed seedlings compared to the wild type.

## Introduction

*Dendrobium officinale* is an important and endangered species. This orchid is valued for its beautiful flower and significant medicinal value (Harriman, 2010; Yongsun and Wenhong, 2011; Xia et al., 2012; Luo et al., 2017). *D. officinale* has a long history of being used as a traditional Chinese medicine (Meng et al., 2013). Modern medicinal research has proven that *D. officinale* contains a number of bioactive substances, such as polysaccharides, phenols, alkaloids, and amino acids that have been widely used to alleviate rheumatoid arthritis, diabetes, obesity, and many other diseases (Xing et al., 2013; Zhao et al., 2016). Among these substances, polysaccharides, which are the most important medicinal substances of *D. officinale*, attract many researchers’ attention. Recently, polysaccharides have been proven to have positive effects on the improvement of antioxidant activity and alleviation of colitis, pulmonary fibrosis, and apoptosis (Huang et al., 2016; Zhang et al., 2017a; Chen et al., 2018a; Liang et al., 2018a). What is more, the bioactivities, composition, structure, and physicochemical properties of polysaccharides from *Dendrobium* have been well defined (Wei et al., 2016; Zhang et al., 2016; Ma et al., 2018), and a number of critical genes related to polysaccharide synthesis have been characterized as well, such as uridine diphosphate glucose pyrophosphorylases (UGPases), sucrose-phosphate synthases (SPSs), sucrose synthases (SuSys), and glycosyltransferases (GTs) including cellulose synthase like A (*CSLA*s) genes (He et al., 2015; Yan et al., 2015; Shen et al., 2017). However, systemic study on the polysaccharide biosynthesis pathway is still limited in *D. officinale*.

GTs are a very widespread group of carbohydrate-active-related enzymes, and participate in glycan and glycoside biosynthesis in higher plants (Shen et al., 2017). Several lines of evidence have demonstrated that the CSLA family plays important roles in the biosynthesis of mannan polysaccharides in many plant species. For example, *CSLA* genes from guar (*Cyamopsis tetragonolobus*), Arabidopsis, and poplar (*Populus trichocarpa*) are capable of producing β-mannan polysaccharides (Dhugga et al., 2004; Liepman et al., 2005; Suzuki et al., 2006; Goubet et al., 2009; He et al., 2015). In higher plants, sucrose metabolism is mainly catalyzed by SPS and SuSy. SPSs promote sucrose synthesis, and UGPases produce glucose-1-phosphate (Glc-1-P) using SuSy-synthesized uridine diphosphate glucose (UDP-Glc). In *D. officinale*, it has been revealed that increased polysaccharide content is directly correlated with the reduced sugar content in the cell, and this process is thought to be mediated by sucrose invertase and SPS. However, this hypothesis requires further investigation (Kleczkowski et al., 2004; Yan et al., 2015). The medicinal values of *D. officinale* mainly depend on the polysaccharide quality and quantity; therefore, more effort is required to elucidate the functions of these genes in *D. officinale*.

UGPases catalyze reversible production of UDP-Glc and Glc-1-P and uridine triphosphate (Kleczkowski, 1994). Studies have shown that UGPases are key enzymes in the synthesis of structural or storage polysaccharides including some active ones such as pullulan (Wu et al., 2002; Kleczkowski et al., 2004; Lian et al., 2012; Zhu et al., 2016; Zhang et al., 2017b). However, studies on UGPase genes (*UGPs*) in *D. officinale* are still limited. It has been known that *DoUGP* is universally expressed in almost all organs especially in the stems of *D. officinale* (Wan et al., 2017). However, does *DoUGP* influence the polysaccharide content or growth of *D. officinale* like *UGP* genes in other plants? If so, are other genes involved in the process? Are there any other functions, such as stress tolerance, related with *DoUGP*? These questions remain to be unanswered.

In this study, to know more about the function of *DoUGP*, *DoUGP* was overexpressed in *D. officinale*, and then, the polysaccharide content, biomass, and stress tolerance were investigated. Polysaccharide content assays indicated that the overexpression of *DoUGP* has a positive effect on *D. officinale* biomass accumulation and stress tolerance. Our study may provide solutions for quality improvement of the important medicinal plant *D. officinale* and contribute to further understanding of the function of the key gene, *DoUGP*, in polysaccharide synthesis.

## Materials and Methods

### Plant Materials and Culture Media

Primary protocorms generated from *D. officinale* seeds were used as explants for the transformations. For sterilization, wild-type protocorms were treated with 75% (*v/v*) ethanol for 30 s followed by the treatment of 0.1%(*w/v*) HgCl_2_ for 5 min. Protocorms were maintained and subcultured on a solidified MS (Murashige and Skoog, 1962) basal salt medium (Phyto Technology Laboratories^®^, Lenexa, KS) containing 30 g L^–1^ of sucrose, 5 g L^–1^ of plant agar (gel strength >1,100 g cm^–2^) (Duchefa Biochemie, Haarlem, Netherlands), 20 g L^–1^ of ground potato, 0.5 mg L^–1^ of 1-naphthaleneacetic acid (NAA), 1 mg L^–1^ of 6-benzylaminopurine (6-BA), and adjusted pH to 5.8. For the ground potato, 20 g of fresh potato tubers were pureed in a blender and then used for medium preparation without filtering. Protocorms were cultured at 25°C with cool fluorescent white light (Panasonic, Osaka, Japan) at an intensity of 70–90 μmol m^2^ s^–1^ and a 14 h photoperiod. These culture conditions were used for all stages of tissue culture except as described below. The co-cultivation medium was prepared by supplementing MS with sucrose, ground potato, and 100 μM acetosyringone (AS) without any plant growth regulators. Selection medium was prepared by supplementing MS medium with 30 g L^–1^ of sucrose, 5 g L^–1^ of agar, 20 g L^–1^ of ground potato, 0.2 mg L^–1^ of NAA, 1 mgL^–1^ of 6-BA, 20 mg L^–1^ of hygromycin B, and 500 mg L^–1^ of cefotaxime, and adjusted pH to 5.8. When the medium was prepared, pH was adjusted with 10% (*w/v*) KOH, all antibiotics were added after autoclaving at 121°C for 20 min, and all growth regulators were added before autoclaving. Except for those reagents described above, all other reagents were provided by Solarbio, Beijing, China.

### Gene Expression Vector Reconstruction and Gene Transformation

**FIGURE 1:**
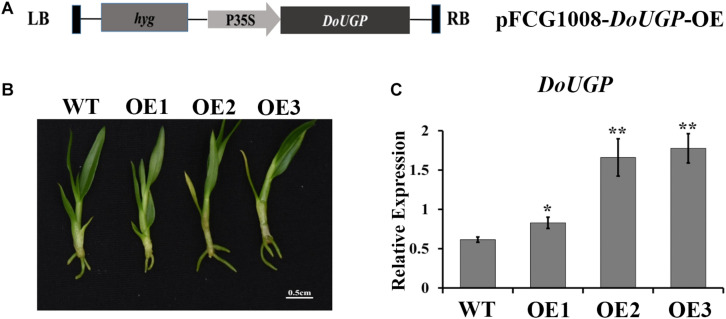
Transformation and verification of *DoUGP* over-expression plants. **(A)** Construction of recombinant expression vector pFGC1008–*DoUGP*–OE. **(B)** Morphology of wild-type and three independent transgenic *DoUGP* overexpression lines (OE1–OE3). **(C)** *DoUGP* mRNA expression levels in wild-type and transgenic lines. Results are presented as mean ± standard error calculated from three biological replicates. Asterisks indicate the statistically significant differences (Student’s *t*-test, *p* < 0.05).

The *DoUGP* overexpression construct (*DoUGP* OE) (Figure 1A) (gene bank ID: KF711982.1) was used for this study. The plasmid was transformed into *Agrobacterium tumefaciens* GV3101 (Chan et al., 1993). When the *A. tumefaciens* culture OD_600_ reached 0.8, the cells were collected by centrifugation at 3,000 × *g* for 10 min. Before infection, the pellets were re-suspended in a liquid MS basal salt and adjusted to OD_600_ of 0.6. Two-month-old protocorms (Supplementary Figure S1) were used for *A. tumefaciens*-mediated transformation. When performing infections, the protocorms were incubated in the *A. tumefaciens* suspension at 28°C for 30 min with agitation at 50 rpm on a shaker. After infection, the protocorms were transferred to filter papers to remove spare liquid.

The infected protocorms were transferred onto the top of a cocultivation medium containing 100 μM AS (Supplementary Figure S1). After cocultivation at 20°C for 4 days, about 30 protocorms per plate were placed on each selection medium (MS supplemented with 30 g L^–1^ of sucrose, 5 g L^–1^ of agar, 20 g L^–1^ of ground potato, 0.2 mg L^–1^ of NAA, 1 mg L^–1^ of 6-BA, 20 mg L^–1^ ^*of*^ hygromycin B, 500 mg L^–1^ of cefotaxime, pH 5.8). After about 60 days, the surviving green transformants were counted and imaged for statistical analysis (Supplementary Figure S1). Then, these protocorms were transferred onto a regeneration medium (MS medium supplemented with 30 g L^–1^ of sucrose, 5 g L^–1^ of agar, 20 g L^–1^ of ground potato, 0.2 mg L^–1^ of NAA, and 1 mg L^–1^ of 6-BA, pH 5.8). For wild-type protocorms, the same medium, but without hygromycin, was used.

### RNA Extraction and Quantitative Real-Time-PCR Analysis

Total RNA from the wild-type and three independent overexpression lines was extracted using 100 μg of 12-month-old plants by using an EASYspin Plus RNA extraction kit (Aidlab, Beijing, China), and cDNA was synthesized using a first-strand cDNA synthesis kit (Thermo, Waltham, MA, United States). Quantitative real-time PCR (qRT-PCR) was performed using SsoFast EvaGreen Supermix and CFX96 (TaKara, Dalian, China) to evaluate the expression levels of *DoUGP* and its metabolic upstream and downstream genes in the wild-type and transgenic plants. Transcript levels were normalized against glyceraldehyde 3-phosphate dehydrogenase (GAPDH). The cDNA was amplified under the following cycling conditions: (1) one cycle of 95°C for 30 s; (2) 40 cycles of 95°C for 15 s, 55°C for 15 s, and 72°C for 15 s; and (3) melt-curve from 65°C to 95°C by 5 s per step with a 0.5°C increment. The sequences of gene-specific primers used for the amplifications are listed in Supplementary Table S1. Results are presented as mean ± standard error calculated from three biological replicates.

### Polysaccharide Extraction and Content Assay

Three grams of shoots from fresh wild-type and two independent transgenic *D. officinale* seedlings were ground with a mortar at room temperature and suspended in 20 ml of distilled water. The suspended samples were heated in a water bath at 80°C for 2 h. Then the supernatant was collected by centrifugation at 6,000 × *g* for 5 min and mixed with 20 ml of distilled water. The sediment was used for further polysaccharide extraction by repeating heating, suspending, and centrifugation operation steps for another two times. The crude polysaccharide solutions from three extractions were mixed well. Ethanol was added to each 5 ml of collected supernatant to the final volume of 25 ml. Then the well-mixed solution was allowed to stand for polysaccharide precipitation at 4°C for 24 h. Precipitate was collected by centrifugation at 6,000 × *g* for 10 min. Collected precipitate was dissolved with hot water (60°C). Then the polysaccharide in the prepared solution was determined by sulfuric acid (80%)–anthrone (0.1%) colorimetry with UV spectrophotometer (UV765, Shanghai, China) at 620 nm, and glucose (0.1 mg L^–1^) was used to build the standard curves (Li et al., 2016). Results are presented as mean ± standard error calculated from three biological replicates.

### Cellulose Content Assay

Cellulose contents were measured, as described previously (Wang et al., 2020). Mature leaf samples (>0.3 g) from 6-month-old wild type and three independent transgenic *D. officinale* seedlings were homogenized with 80% (v/v) ethanol with a mortar at room temperature followed by centrifugation. Then the crude extraction was digested with sulfuric acid. Cellulose contents were determined by using a cellulose detection kit (Suzhou Comin biotechnology, Suzhou, China). Results are presented as mean ± standard error calculated from three biological replicates.

### Analysis of Monosaccharides Composition of the Polysaccharides

Stems of 18-month-old wild-type and three independent transgenic *D. officinale* seedlings were dried at 80°C until constant weight. Then the dried stems were grounded to powder with a mortar at room temperature. Around 0.3 g of dry powder was used to extract water-soluble polysaccharides, which were then analyzed by using high-performance liquid chromatography (HPLC) according to the reported method (He et al., 2018). Briefly, the powder of each sample was pre-extracted with 80% ethanol at 80°C for 2 h. This process was repeated twice to further remove monosaccharides, oligosaccharides, and ethanol-soluble materials. Then, the water-soluble polysaccharides were extracted with double-distilled water at 100°C for 2.5 h. The extraction was hydrolyzed with 3 M HCl, derivatized with 1-phenyl-3-methyl-5-pyrazolone (PMP), and monosaccharide content was analyzed by using HPLC.

### Quasi-Targeted Metabolomics Analysis of Monosaccharide Composition of Soluble Polysaccharide

Metabolite extraction, HPLC-MS/MS analysis, metabolite identification and quantification, and data analysis were performed according to the protocol of the laboratory (Novogene Bioinformatics Technology Co., Ltd.). Briefly, 100 mg stems were collected and ground with liquid nitrogen. Then the homogenate was resuspended with prechilled 500 μl of 80% ethanol and 0.1% formic acid by well vortexing. After being centrifugated at 15,000 rpm at 4°C for 10 min, the supernatant was diluted to the final concentration containing 53% methanol and injected into the LC-MS/MS system for analysis. HPLC-MS/MS analyses were carried out using an ExionLC AD system (SCIEX) coupled with a QTRAP 6500+ mass spectrometer (SCIEX) in positive and negative ion modes, respectively. Metabolite identification was based on Q1, Q3, retention time, decluttering potential, and collision energy, and Q3 was used for metabolite quantification. The original data files were processed using the SCIEX OS Version 1.4 to integrate and correct the peak. These metabolites were annotated using the KEGG database^1^, HMDB database^2^, and Lipidmaps database^3^.

### Freezing and Osmotic Stress Treatments

To determine the effects of different stress on transgenic *D. officinale*, plants of wild-type and three independent overexpression lines with a height of 2–3 cm were selected and cultured in a medium supplemented with mannitol (500 mM) for osmotic stress treatment. Freezing treatment was performed by treating plants at −20°C for 1 h. Ten or Twenty days after freezing or osmotic treatment, plants were harvested for chlorophyll and proline content and SOD enzyme activity tests. Results are presented as mean ± standard error calculated from three biological replicates.

### Determination of Chlorophyll and Proline Contents and Superoxide Dismutase Activities

To test the chlorophyll content, fully expanded leaf samples from fresh wild-type and three independent transgenic *D. officinale* seedlings were homogenized with 95% (v/v) ethanol with a mortar at room temperature followed by centrifugation. The absorbance was measured at 665 and 649 nm for chlorophyll *a* and chlorophyll *b*, respectively, using an ultraviolet spectrophotometer (UV765, Shanghai, China). Proline content was determined by using the acid-ninhydrin method, as described previously, with modifications (Tavakoli et al., 2016). SOD activity was assayed by measuring the inhibition of the photochemical reduction of NBT. Chlorophyll, proline, and SOD contents were determined by using materials from three independent transgenic seedlings. Results are presented as mean ± standard error calculated from three biological replicates.

### Statistical Analysis

All data collected from the experiments were subjected to a Student’s *t*-test using Microsoft Excel (Microsoft Corporate, Remond, WA, United States).

## Results

### Construction of *DoUGP* Overexpression *Dendrobium officinale* Seedlings

To examine the function of *DoUGP* in *D. officinale*, in this study, we generated *DoUGP* overexpression of *D. officinale* seedlings. The *DoUGP*-OE construct, which is composed of a CaMV 35S-driven *DoUGP* cDNA (Figure 1A), was introduced into *D. officinale* genome and transformation frequency was up to 16.9%. To confirm whether the *DoUGP* was indeed overexpressed, the mRNA level of *DoUGP* was determined by using qRT-PCR. Three lines (Figure 1B) with increased mRNA expression levels from 1.35- to 2.89-fold were selected for further study (Figure 1C). Glc-1-P and UDP-Glc are the substrate and product of DoUGPase-mediated reaction. Metabolomics data showed that Glc-1-P was slightly decreased, and UDP-Glc was significantly increased, in the transgenic plants. This result suggests that the overexpressed *DoUGP* resulted in an increased UGPase activity (Supplementary Figure S2). These three transgenic lines exhibited different expression levels, which could be utilized for the analysis of the overexpression of *DoUGP* in *D. officinale.*

### Overexpression of *DoUGP* Promoted *Dendrobium officinale* Biomass

**FIGURE 2:**
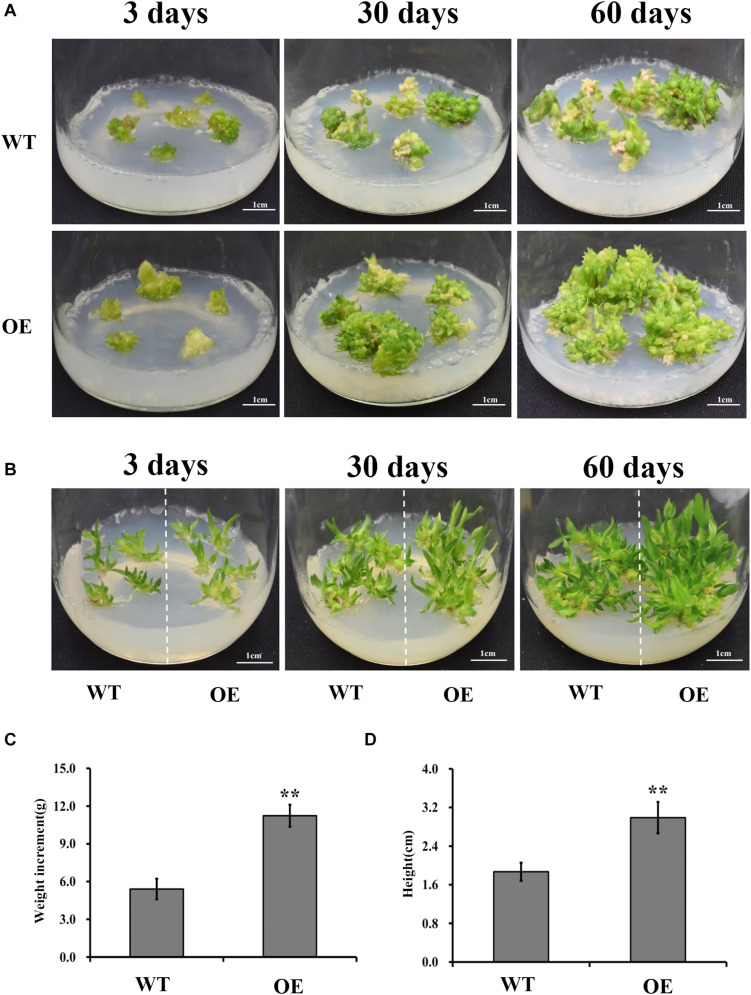
Growth performance of *DoUGP* overexpression (OE3) plants. Protocorms **(A)** and seedlings **(B)** were imaged when they were cultivated on MS medium for 3, 30, and 60 days, respectively. For weight **(C)** measurement, during 60 days, weight increment of protocorms from three independent culture flasks were investigated. For height **(D)** measurement, seedlings from three independent culture flasks were investigated after 60 days. Results are presented as mean ± standard error calculated from three biological replicates. Asterisks indicate statistically significant differences.

Studies have shown that UGPases, along with other polysaccharide synthesis-related enzymes, promote synthesis of structural or storage polysaccharides (Coleman et al., 2006; Wang et al., 2011). If *DoUGP* has a similar function as the other UGPases, increased biomass is expected in *DoUGP* overexpression transgenic seedlings. Therefore, we investigated the growth of transgenic and wild-type *D. officinale* plants at different stages (3, 30, and 60 days after transplanting) by measuring the biomass of protocorms, and seedlings were determined. Interestingly, both higher growth rate and increased biomass of protocorms were observed in the transgenic seedlings compared to the wild-type ones after 3, 30, and 60 days after transplanting (Figure 2A and Supplementary Figure S3). Similar results were observed in the wild-type and transgenic seedlings on 3, 30, and 60 days after transplanting (Figure 2B and Supplementary Figure S3). Moreover, the weight of protocorms and the height of seedlings were around twofold of the corresponding ones of wild-type (Figures 2C,D) after 60 days. Based on these results, we concluded that the overexpression of *DoUGP* may increase the biomass of *D. officinale* at both the protocorm and seedling stages.

### Overexpression of *DoUGP* Promoted Expression Levels of the Up-and Down-Stream Genes in Starch and Sucrose Metabolism Pathway

**FIGURE 3:**
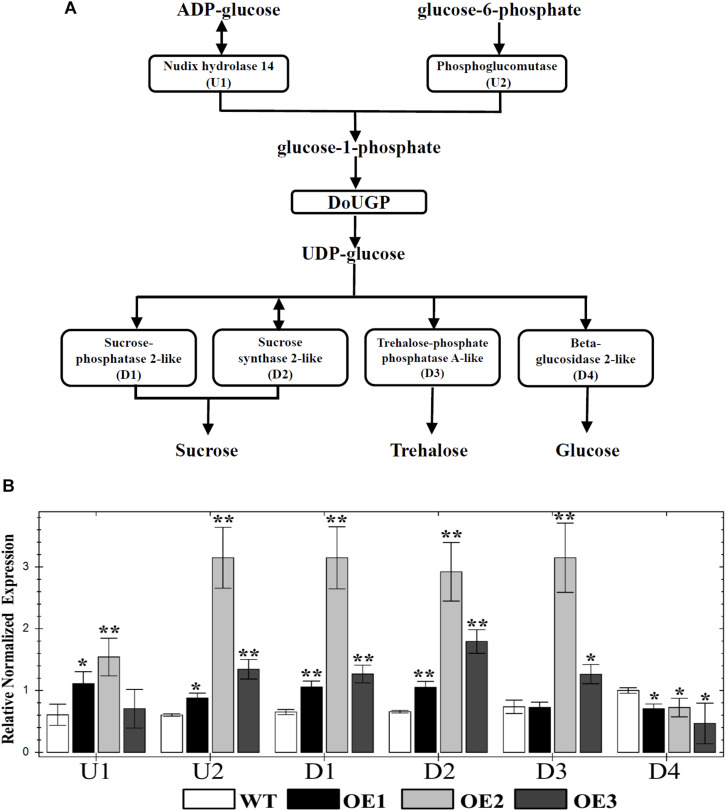
Quantitative real-time (qRT)-PCR analysis of up/downstream genes of *DoUGP* in starch and sucrose metabolism pathway. **(A)** Starch and sucrose metabolism pathway and critical genes. **(B)** Relative expression levels of up/downstream genes of *DoUGP* in leaves of transgenic and wild-type plants. Results are presented as mean ± standard error calculated from three biological replicates. Asterisks indicate the statistically significant differences (Student’s *t*-test, *p* < 0.05).

It has been well studied that *UGP* genes play roles in the sucrose and starch biosynthesis pathway (Kleczkowski, 1994). UGPases catalyze the production of UDP-glc and pyrophosphate from Glc-1-P and uridine triphosphate (Kleczkowski, 1994). In some cases, increased activity of a single enzyme may have an effect on the up- and downstream enzyme activities or gene expression levels of other members of a pathway. However, in other cases, the increased enzyme activities may not affect the expression levels of the genes in the same pathway. To further investigate whether the metabolism-related genes are affected by *DoUGP* overexpression, we chose several critical genes (U1, nudix hydrolase 14; U2, phosphoglucomutase; D1, sucrose-phosphatase 2-like; D2, sucrose synthase 2-like; D3, trehalose-phosphate phosphatase A-like and D4, beta-glucosidase 2-like) through the KEGG database^4^. The positions of these genes in the pathway related to *DoUGP* are shown in Figure 3A. qRT-PCR results showed that except D4, which was slightly decreased, the other genes were all dramatically increased in all three transgenic lines compared to the ones in the wild-type plants (Figure 3B). These results imply that the overexpressed *DoUGP* alters the expression of the genes in the same pathway, thereby, overexpressed *DoUGP* affects the pathway.

### Overexpression of *DoUGP* Increased Soluble Polysaccharide Content in *Dendrobium officinale*

**FIGURE 4:**
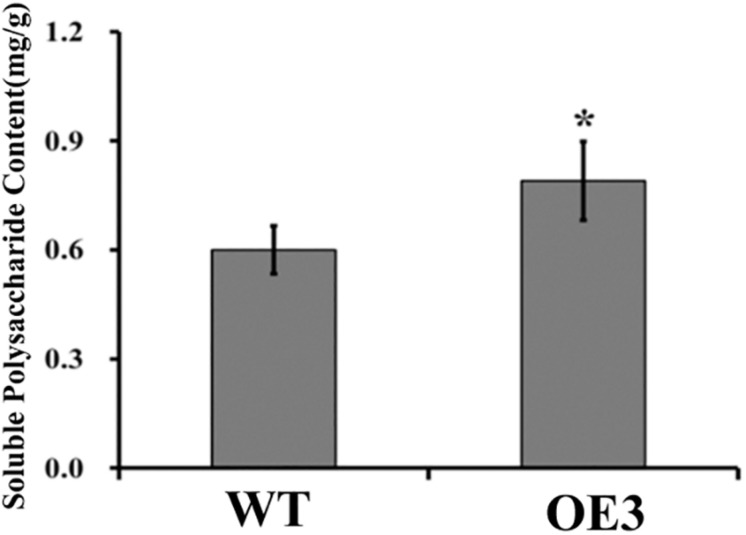
Comparison of total soluble polysaccharide contents in wild-type (WT) and *DoUGP* overexpresssion (OE3) plants. Results are presented as mean ± standard error calculated from three biological replicates. Asterisk indicates statistically significant differences (Student’s *t*-test, *p* < 0.05).

Soluble polysaccharide content is an important index for judging the medicinal value of *D. officinale*. As both *DoUGP* and *CSLAs* play important roles in the polysaccharide synthesis and in the stem of transgenic plants with overexpressed *DoUGP*, most *CSLA*s’ expression was significantly increased, and it is a reasonable hypothesis that polysaccharide content in the transgenic plants may be affected as well. To verify this hypothesis, the polysaccharide content of wild-type and transgenic plants was determined. When *DoUGP* was overexpressed, polysaccharide content was increased by 11.1% (Figure 4 and Supplementary Figure S4). Metabolomics data of monosaccharide composition of water-soluble polysaccharides in the stem of wild-type and transgenic *D. officinale* plants show that mannose is mainly responsible for the increased polysaccharide content in the *DoUGP* overexpression plants (Supplementary Table S1). However, the glucose ratio of monosaccharide composition in the transgenic plants was less compared to the one in the wild-type plants (Supplementary Table S1). These data further confirmed that *DoUGP* plays important roles in the polysaccharide synthesis network. *UGP*s have been reported to play roles in the cellulose synthesis pathway (Zhang et al., 2013). However, overexpressed *DoUGP* decreased the cellulose content in the transgenic plants (Supplementary Figure S5).

### Overexpression of *DoUGP* Had an Effect on Cellulose Synthase-Like A Gene Family Expression in Mannan Polysaccharides Biosynthesis Pathway

**FIGURE 5:**
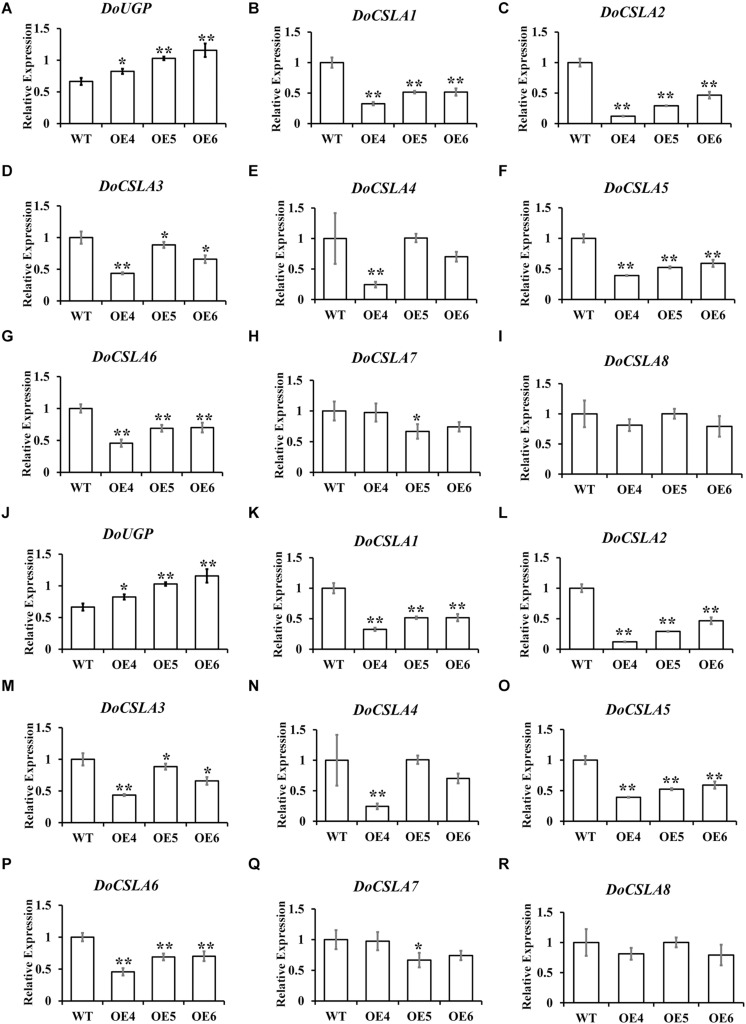
Expression levels of cellulose synthase-like A (*CSLA*) family genes in stems **(A–I)** and leaves **(J–R)** of *DoUGP* overexpression plants. Results are presented as mean ± standard error calculated from three biological replicates. Asterisks indicate the statistically significant differences (Student’s *t*-test, *p* < 0.05).

It has been proven that mannose is a major component of polysaccharides in *Dendrobium* species (Meng et al., 2013), and eight cellulose synthase-like A genes (CSLAs), which are likely related to the biosynthesis of bioactive mannan polysaccharides, have been identified in *D. officinale* (Shen et al., 2017). As the overexpression of *DoUGP* disturbed the expression of genes in starch and sucrose pathways, we wonder if these eight genes would be affected by the overexpression of *DoUGP* as well. Therefore, expression patterns of these eight *CSLA* genes in *DoUGP* overexpression plants were determined by qRT-PCR. Expression of all *DoCSLA* genes except *DoCSLA8* significantly increased in the stem of *DoUGP* overexpression plants (Figures 5A–I). However, to our surprise, the expression of all *DoCSLA* genes except *DoCSLA8* were decreased expression in the leaves of *D. officinale* (Figures 5J–R). These results suggest that by affecting the expression of *DoCSLA* genes, DoUGPase or DoUGPase-mediated metabolism may play important roles in the mannan polysaccharide synthesis network.

### Overexpression of *DoUGP* Enhanced Stress Tolerance in *Dendrobium officinale*

**FIGURE 6:**
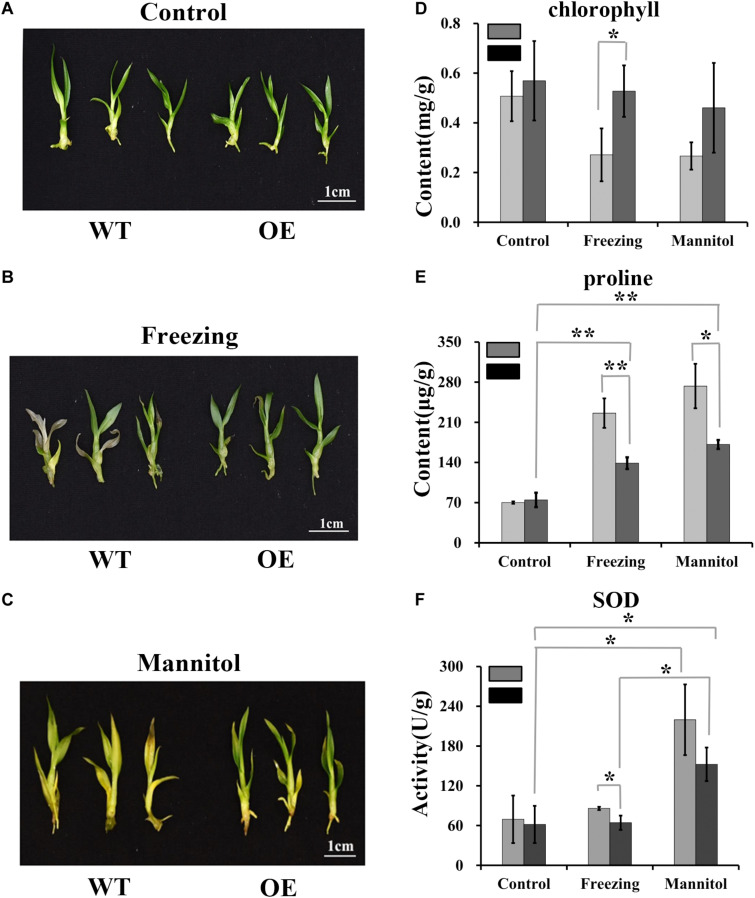
Resistance of *DoUGP* overexpression plants to freezing and osmotic stress treatments. **(A)** Wild-type (WT) and *DoUGP* overexpression (OE) seedlings cultured without stress. **(B)** Wild-type (WT) and *DoUGP* overexpression seedlings treated at -20°C for 1 h and cultured for another 10 days. **(C)** Wild-type (WT) and *DoUGP* overexpression seedlings treated with 500 mM mannitol for 20 days. **(D)** Chlorophyll contents of wild-type (gray bar) and *DoUGP* overexpression (black bar) seedlings after freezing or osmotic stress treatments. **(E)** Proline contents of wild-type and *DoUGP* overexpression seedlings after freezing or osmotic stress treatments. **(F)** SOD activities after freezing or osmotic stress treatments. Chlorophyll, proline, and SOD contents were determined by using mixed materials from three independent transgenic seedlings. Results are presented as mean ± standard error calculated from three biological replicates. Asterisks indicate the statistically significant differences (Student’s *t*-test, *p* < 0.05).

High polysaccharide content may enhance plant stress tolerance (Livingston et al., 2009; Correa-Ferreira et al., 2019; Liu et al., 2019; Zou et al., 2019). To determine whether *DoUGP* overexpression may confer increased stress tolerance to plants, we performed stress treatments using wild-type and transgenic plants. Freezing or osmotic stress treatment were conducted by treating the wild-type and transgenic plants with -20°C or 500 mM mannitol. Chlorophyll and proline contents and superoxide dismutase (SOD) activity were determined. Compared to the chlorophyll content of wild-type plants, after freezing treatment, the chlorophyll content of transgenic plants was 0.67-fold higher (Figure 6D). Besides, proline content decreased 0.63-fold, and there was a 0.34-fold decrease in superoxide dismutase (SOD) activities detected in the transgenic plants after freezing treatments (Figures 6E,F). After osmotic stress, a 0.59-fold reduced proline content was also detected in the transgenic plants (Figure 6E). These results suggest that the overexpressed *DoUGP* may enhance plant stress tolerance by increasing the polysaccharide content (Figures 6A–C).

## Discussion

*D. officinale* is a traditional but endangered Chinese orchid. As an important component of *D. officinale*, polysaccharide has significant positive effects such as antioxidation, acute colitis, pulmonary fibrosis, and apoptosis (Zhao et al., 2017; Chen et al., 2018a; Liang et al., 2018b, 2019). In order to improve the polysaccharide yield of *D. officinale*, agricultural and polysaccharide processing approaches such as fertilizer application and optimized polysaccharide extraction and dehydration methods have been applied (He et al., 2018; Qian et al., 2018). However, so far, no satisfactory results have been achieved. Since some genes, such as *UGP*, *SPS*, *SuSy*, *GTs*, and *CSLA* (He et al., 2015; Yan et al., 2015; Shen et al., 2017), related to polysaccharide synthesis have been characterized, enhancing the expression of these genes may be a direct and efficient solution for polysaccharide yield and quality improvement in *D. officinale*. Therefore, in this study, we overexpressed the *DoUGP* gene of *D. officinale* aiming to obtain a higher polysaccharide content in transgenic plants. To our surprise, besides the expected polysaccharide content increase, the transgenic plants exhibited improved biomass and stress tolerance, as well, which are important agricultural traits. These phenotypes have not been found in transgenic plants only containing a vector (Li et al., 2017; Chen et al., 2018b). Therefore, we are confident that the increased biomass and enhanced stress tolerance, along with the increased polysaccharide contents, were due to the *DoUGP* overexpression. We suppose these improvements were due to the accumulated polysaccharide, which promotes plant growth and enhances osmotic pressure tolerance.

In this study, we also found that when *DoUGP* was overexpressed, some of the key genes in the same pathway were significantly affected. Surprisingly, among the four downstream genes, three of them were upregulated; nevertheless, beta-glucosidase 2-like gene was downregulated (Figure 3B). This result indicates that these four genes may have other functions in different ways at least when working along with *DoUGP*. We also noticed that upregulation of these genes such as nudix hydrolase 14 gene and sucrose phosphatase 2-like gene was not consistently related to *DoUGP* overexpression levels. This phenomenon could be explained by the regulation of nudix hydrolase 14 and sucrose phosphatase 2-like genes by *DoUGP* that was in a fine tuned way. Moreover, we also noticed that in the stem, most of the *CSLA* genes in the mannan polysaccharide synthesis pathway were upregulated (Figures 5A–I), which is consistent with the increased total soluble polysaccharide content (Figure 4). Besides, the increased expression of most of the *CSLA* genes is also consistent with the promoted ratio of mannan to total soluble polysaccharide in the stem (Supplementary Table S1). Although the monosaccharide composition of water-soluble polysaccharide data indicated that the relative ratio of glucose in the DoUGP-OE transgenic plants was decreased compared to the one of WT, given the increased polysaccharide content resulting by the overexpressed *DoUGP*, the absolute glucose content in the transgenic plants was not less than the one of WT. However, in the leaves of *DoUGP*-OE transgenic plants, the expression of *CSLA* genes was significantly downregulated, which indicates that the overexpression of *DoUGP* affected the *CSLA* gene expression in a tissue-dependent way (Figures 5J–R). This implies that in the *D. officinale*, the polysaccharide metabolism network is complex and requires more effort to elucidate it.

The reason we started with *DoUGP* is to learn about the underlying mechanisms of polysaccharide synthesis in *D. officinale* and apply this knowledge to improve the polysaccharide content in these plants. In other plants, the *UGP*s have been reported to play roles in the cellulose synthesis pathway (Zhang et al., 2013). However, in our case, instead of an increase in cellulose content, overexpressed *DoUGP* resulted in the increased total soluble polysaccharides (Supplementary Figure S5). This result indicates that studies on *DoUGP* are important for *D. officinale*, as the soluble polysaccharide content is critical for the medicinal use of *D. officinale*. For medicinal use, *D. officinale* plants of 2–4 years old are regarded most appropriate for polysaccharide extraction, as polysaccharide content of *D. officinale* varies depending on age and developmental stages (Qin et al., 2018). *D. officinale* plants used in this study were 1-year-old young plants, which means a much higher polysaccharide content could be expected in 2–4-years-old transgenic plants. We also noticed that the stress tolerance of transgenic plants was significantly improved possibly due to the increased polysaccharide content (Figure 6).

**FIGURE 7:**
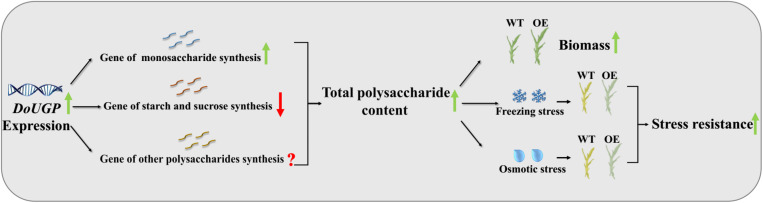
Schematic diagram of how *DoUGP* overexpression enhances biomass and promotes stress resistance of *D. officinale* plants.

Based on the results obtained in this study, a schematic map was summarized (Figure 7). First, when *DoUGP* was overexpressed, some genes in the same synthesis pathway were promoted. As a result, the total polysaccharide content was increased in the transgenic plants. Besides these two polysaccharide synthesis pathways, the gene expression of other polysaccharide synthesis pathways remains unclear. Increased polysaccharide content, enhanced biomass accumulation, and improved stress resistance were obtained in *DoUGP* overexpression of *D. officinale* plants. All these traits are critical features for *D. officinale* breeding. We suppose that increased freezing and osmotic resistance resulting from increased polysaccharide content may be due to a better maintained intracellular osmotic pressure and freezing point. Besides, decreased proline content and SOD enzyme activity suggest that the relieved stress in the transgenic plants is due to the presence of additional polysaccharide content.

## Data Availability Statement

All datasets generated for this study are included in the article/Supplementary Material.

## Author Contributions

JC, JH, and MT conceived and designed the experiments and provided funding for research work. LW and JC performed most of the experiments. LW, JH, and JC wrote the manuscript. JH, KZ, FL, and MT analyzed and commented on the data and manuscript. HL, XJ, JW, and FL performed some of the experiments. All authors contributed to the article and approved the submitted version.

## Conflict of Interest

The authors declare that the research was conducted in the absence of any commercial or financial relationships that could be construed as a potential conflict of interest.

## Footnotes

1 http://www.genome.jp/kegg/

2 http://www.hmdb.ca/

3 http://www.lipidmaps.org/

4 https://www.kegg.jp/kegg/kegg3a.html
