# Supplementary material for: Overexpression of DoUGP Enhanced Biomass and Stress Tolerance by Promoting Polysaccharide Accumulation in Dendrobium officinale
Source: Front Plant Sci. 2020 Nov 16;11:533767. doi: 10.3389/fpls.2020.533767 (PMC7703667; doi:10.3389/fpls.2020.533767)
Supplement: Supplementary file 3 [file Data_Sheet_1.DOCX]

**Supplementary Figures**

**
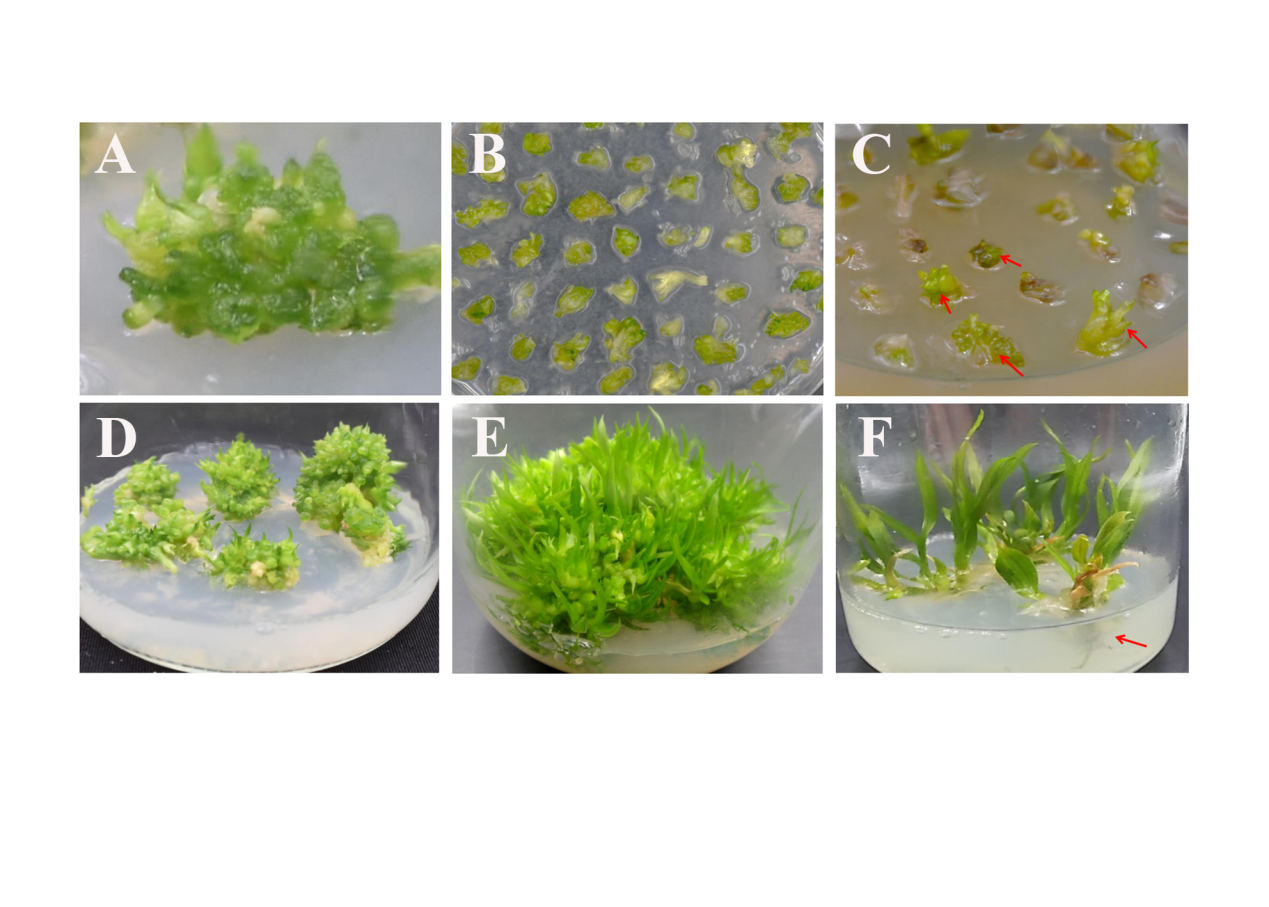
**

**Supplementary Figure S1.** Transformation and selection of *Dendrobium officinale* protocorms with *DoUGP* gene.

(A) Primary protocorms of *Dendrobium officinale*. (B) Protocorms co-cultured with Agrobacteria containing *DoUGP* gene. (C) Infected protocorms selected on hygromycin media. (D) Regenerated protocorms after transformation and selection. (E) Seedling regenerated from transformed protocorms. (F) The intact plantlets regenerated from transformed protocorms. Arrows in panel C and D indicate hygromycin-resistant protocorms and plant roots, respectively.


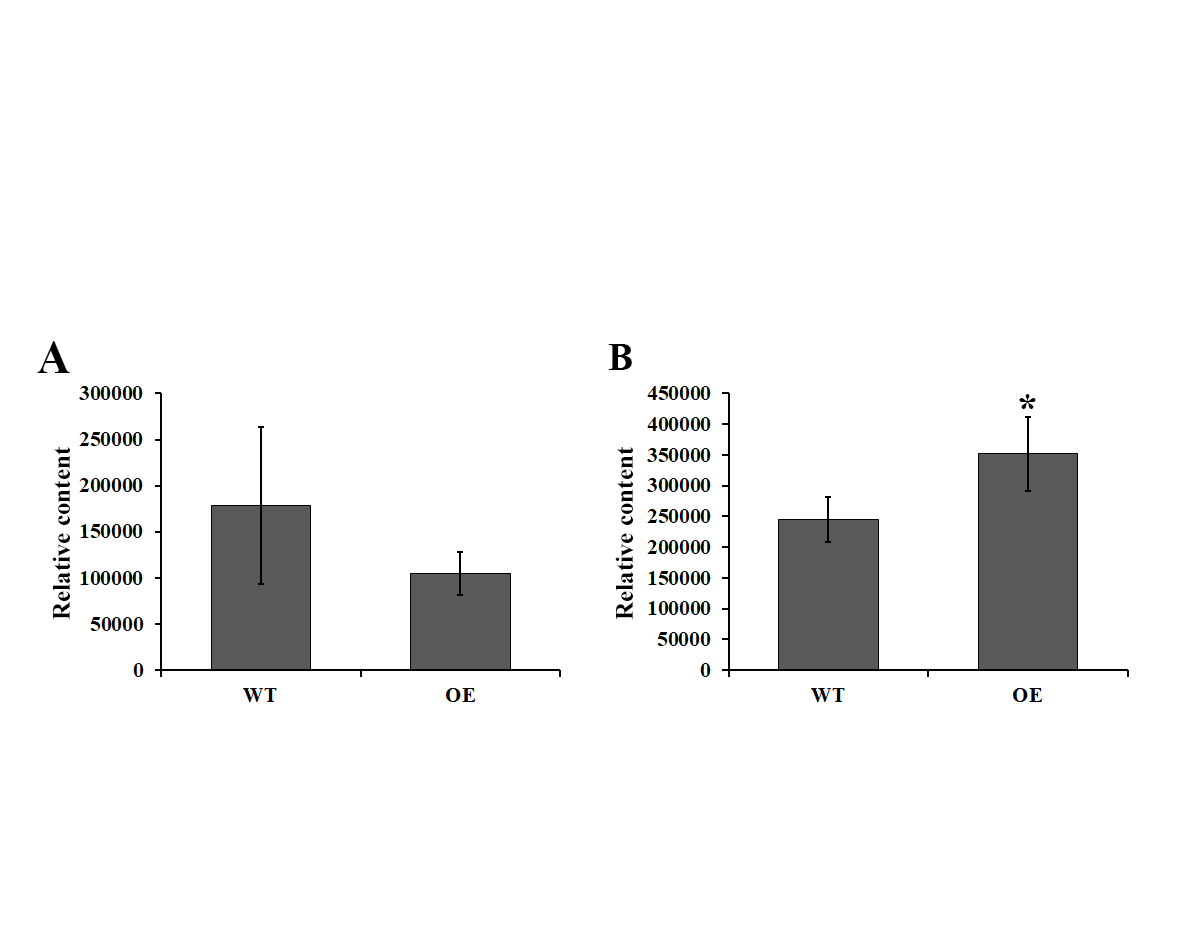


**Supplementary Figure S2.** Comparison of glucose-1-phosphate (A) and uridine diphosphate glucose (B) contents in wild-type (WT) and DoUGP overexpresssion (OE3) plants.

Results are presented as mean ± standard error calculated from three biological replicates. Asterisk indicates the statistically significant differences (Student’s t-test, p < 0.05)


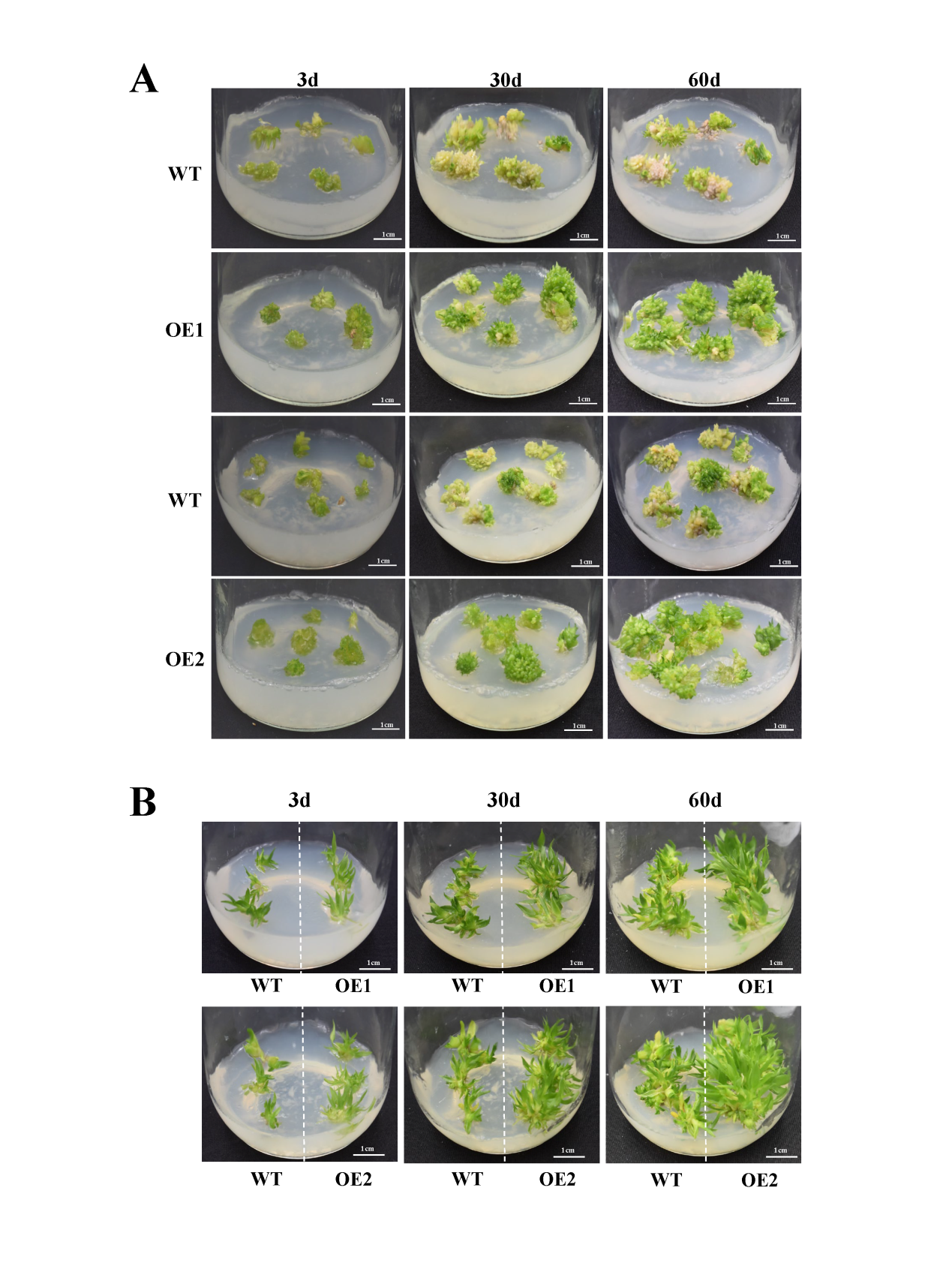


**Supplementary Figure S3.** Growth performance of another two independent *DoUGP* overexpression lines besides OE3.

Protocorms (A) and seedlings (B) of two independent overexpression lines (OE1 and OE2) were imaged when they were cultivated on MS medium for 3 days, 30 days and 60 days，respectively.

**Supplementary Figure S4.** Comparison of total soluble polysaccharide contents in stems of wild-type (WT) and *DoUGP* overexpresssion (OE2) plants.

Results are presented as mean ± standard error calculated from three biological replicates. Asterisk indicates the statistically significant differences (Student’s *t-test*, *p* < 0.05).


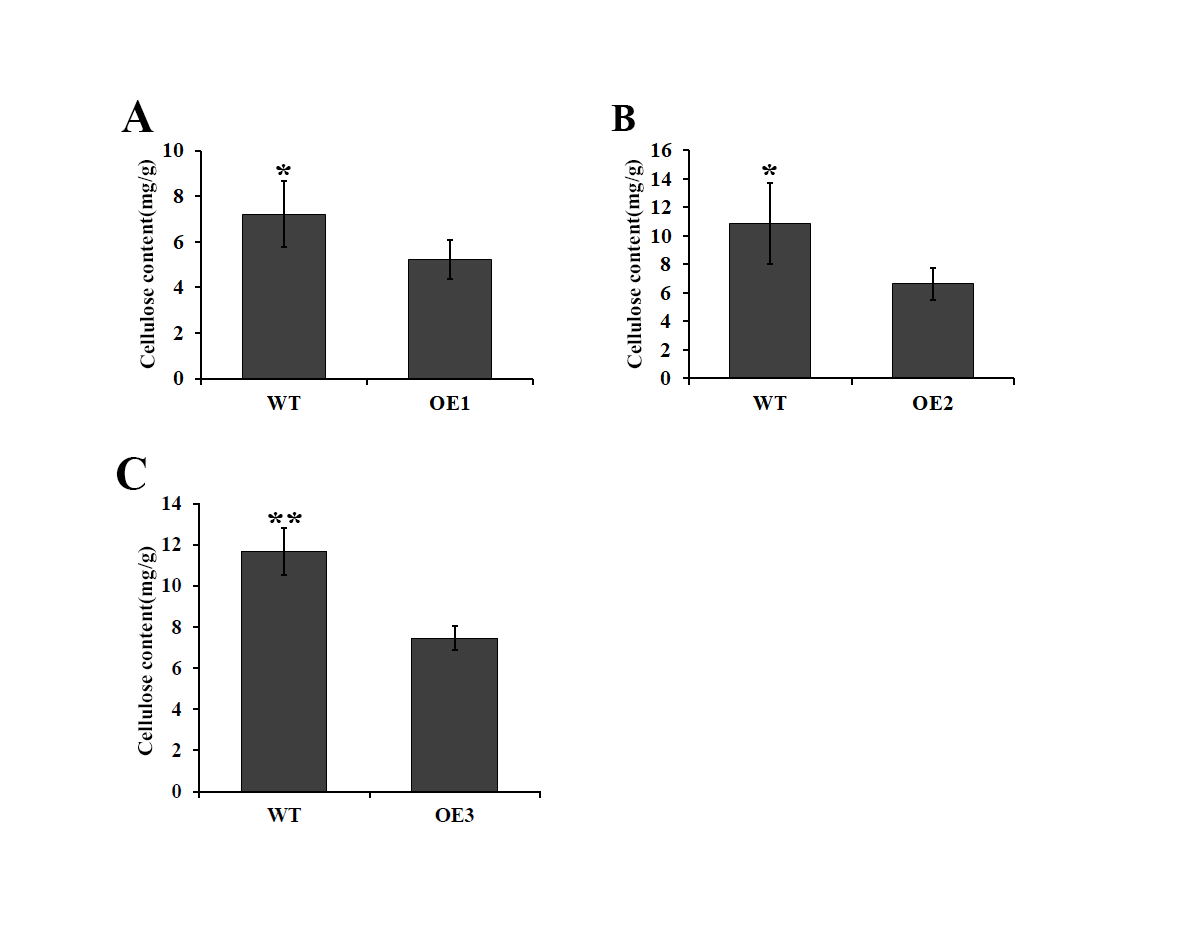


**Supplementary Figure S5.** Comparison of cellulose contents in wild-type (WT) and DoUGP overexpresssion (OE1, OE2 and OE3) plants.

Results are presented as mean ± standard error calculated from three biological replicates. Asterisk indicates the statistically significant differences (Student’s t-test, p < 0.05)
